# The Reorganisation of the University of London

**Published:** 1898-01-29

**Authors:** 


### THE REORGANISATION OF THE UNIVERSITY OF LONDON.

Londoners cannot too quickly or too thoroughly realise the way in which their interests are being played with in regard to the question of establishing a true University of London. At the meeting of the London University Convocation, held last week, it was gravely proposed to submit the scheme for the reconstitution of the University to the whole mass of members. Mr. Fitch very properly pointed out that this meant an indefinite postponement of the measure, and, fortunately, the proposal was negatived. But what Londoners must try to realise is the fact that the members of the present so-called London University are not Londoners, have no interest in London or its wants and have no right whatever to intervene to prevent London getting what it so urgently requires. Who are these people, and what rights do they possess? They are about 4,000 in number, they are scattered all over the globe, they are not Londoners in any sense; they were not even educated in London; they are not, like the members of the older Universities, scholars educated at the same seats of learning, and sharers in the same academic traditions and associations. "The only link," says Mr. Fitch, "which binds them together is the accident that at one period of their lives they were examined by the authorities at Burlington House." The northern towns have combined, and now have their University. Birmingham is putting forward a claim for like privileges, while London remains entirely unprovided for, and that, to a large extent, for the ridiculous reason that about sixty years ago the title of University of London was appropriated by a mere examining and degree-giving body, which, although not possessing or aspiring to possess the functions of a University, stands, like a dog in the manger, in the way of all progress. If Londoners were sincere in their desire for higher education, and if they possessed a scrap of local pride, such as exists in provincial towns, they would with one voice demand the immediate passing of the proposals contained in the London University Commission Bill, 1897, or even of some still stronger measure. If the London University cannot be reconstituted, it should be made to disgorge its title, which it practically stole from University College on the day of its birth in 1836, for it is to a large extent the fact that this heterogeneous and cosmopolitan crowd has bagged the title, and holds on to it with selfish pertinacity, that stands in the way of Londoners getting the University they require.

